# Childhood Trauma and Mental Health Status in General Population: A Series Mediation Examination of Psychological Distress in COVID-19 Pandemic and Global Sleep Quality

**DOI:** 10.3389/fpsyt.2021.782913

**Published:** 2021-12-02

**Authors:** Min Xie, Yiguo Tang, Ling Zhu, Minhan Dai, Yulu Wu, Yunqi Huang, Yunjia Liu, Liling Xiao, Tao Li, Qiang Wang

**Affiliations:** ^1^Mental Health Centre and Psychiatric Laboratory, West China Hospital, Sichuan University, Chengdu, China; ^2^Department of Clinical Psychology, Southwest Hospital, The First Hospital Affiliated to Army Medical University, Third Military Medical University, Chongqing, China; ^3^Affiliated Mental Health Centre and Hangzhou Seventh People's Hospital, Zhejiang University School of Medicine, Hangzhou, China

**Keywords:** coronavirus disease 2019, sleep quality, childhood trauma, depression, anxiety

## Abstract

**Background:** Coronavirus-2019 (COVID-19) has been coexisting with humans for almost 2 years, consistently impacting people's daily life, medical environment, and mental health. This study aimed to test the series mediation model triggered by childhood trauma, in which perceived psychological impact of COVID-19 pandemic and sleep quality mediated the path sequentially and led to adverse mental health outcomes.

**Methods:** A cross-sectional design involving 817 participants were enrolled *via* WeChat online survey. Participants completed questionnaires, including demographic features, the Childhood Trauma Questionnaire, Impact of Event Scale-Revised (IES-R) questionnaire, Pittsburgh Sleep Quality Index (PSQI) questionnaire, and Depression, Anxiety, and Stress Scale (DASS-21). Pearson correlations and hierarchical multiple linear regression were employed to examine the association of childhood trauma and psychological stress of COVID-19, sleep quality, and mental health status. In addition, a series mediate analysis was carried out to examine sequence mediating effects of psychological impact of COVID-19 and sleep quality between childhood trauma and mental health status.

**Results:** The results showed that childhood trauma is positively and significantly related to psychological distress of COVID-19 pandemic, sleep quality, and mental health status (*p* < 0.05). Hierarchical multiple linear regression analysis shown that demographic features explained 4.4, 2.1, and 4.0% of the total variance in DASS-21, IES-R, and PSQI total scale scores, respectively. Adding childhood trauma significantly increased the model variance of DASS-21 (Δ*R*^2^ = 0.129, *F* = 126.092, *p* = 0.000), IES-R (Δ*R*^2^ = 0.062, *F* = 54.771, *p* = 0.000), and PSQI total scale scores (Δ*R*^2^ = 0.055, *F* = 48.733, *p* = 0.000), respectively. Moreover, the series mediation model showed that the perceived impact of the COVID-19 pandemic and sleep quality were sequential mediators between childhood trauma and mental health status (proportion explained: 49.17%, *p* < 0.05).

**Conclusion:** Amid the ravages of COVID-19, childhood trauma predicts poor mental health status, in part because of greater psychological impact related to COVID-19 and poorer global sleep quality. In order to improve mental health, future researchers should pay more attention to individuals with childhood trauma, for its association with greater stress related to life events and poorer sleep quality.

## Introduction

Globally, 2019 coronavirus disease (COVID-19), a highly infectious and potentially fatal disease, has been coexisting with humans for almost 2 years. Previous studies found that COVID-19 has a sudden and massive impact on freedom of movement, daily activity, and medical environment, which could significantly ruin mental health of medical staff, patients with mental disorder, and the general population (1–5). What is more, one meta-analysis found that the overall prevalence of sleep disturbances, depression, and anxiety among COVID-19 patients is 34, 45, and 47%, respectively (6). One study across geographic regions worldwide reported that events related to COVID-19 were more likely to be associated with mental health symptoms, especially symptoms of post-traumatic stress disorder (PTSD), insomnia, depression, and anxiety in the general population (7).

Childhood trauma, emotional or physical adverse experiences in one's early life, was associated with increased risk for developing almost all mental disorders, including sleep disorders, depression, anxiety, bipolar disorder, PTSD, and schizophrenia (8–10). The extant literature suggests that stress exposure during early life may lead to excessive glucocorticoid release, dysfunction of hypothalamic-pituitary-adrenal (HPA) axis, abnormal development of brain trajectories, and changes of epigenetics regulation (11–14). The trauma-psychosis cycle proposed that individual exposures to environmental stressors during early life further impaired their adaptive coping strategies and thus increased the vulnerability to future stressors (15). Stress-sensitization model proposed that dysregulation of stress response caused by exposure to childhood trauma may render an individual more susceptible to psychosis triggered by later stressors (16).

Some previous studies have reported that historical trauma (physical and emotional trauma over the life span and across generations), childhood abuse, and social support were closely related to psychological stress, sleep quality, and emotion regulation during the COVID-19 outbreak (17–19). One study found that psychological stress of COVID-19 mediated the association between childhood trauma and Pittsburgh Sleep Quality Index (PSQI) global sleep quality (18). Another study found that the relationship between childhood trauma and the severity of depressive/anxiety symptoms was partly mediated by insomnia symptoms in severe mental disorders (20). However, our understanding of how traumatic stress symptoms related to COVID-19 and global sleep quality mediate the association between early life trauma and mental health symptoms is currently limited. So, we aim to (1) examine the association between childhood trauma and psychological impact related to COVID-19, sleep quality, and the mental health condition in the general population and (2) explore whether there are mediating effects of traumatic symptoms related to COVID-19 and global sleep quality between childhood trauma and mental health status.

## Methods

### Design and Participants

A cross-sectional survey-based study was conducted from August 20, 2021 to September 5, 2021. All participants in this study were recruited *via* WeChat, the most widely used social media platform in China, and all data were collected using electronic questionnaires *via* online survey tool, Wenjuanxing platform (https://www.wjx.cn/app/survey.aspx). In order to obtain more participation from different regions of China, direct online and snowball recruitment methods through “Circle of Friends” of WeChat were used. There were 948 participants who voluntarily filled in and submitted the questionnaire, among which 52.89, 9.15, 8.83, 5.68, 4.31, 2.84, 2.63, and 2.52% were from Sichuan, Chongqing, Henan, Hebei, Liaoning, Beijing, Jiangsu, and Guangdong, respectively. Our inclusion criteria were as follows: (1) aged from 16 to 60 years old and (2) were able to use smart phones and complete questionnaires. Invalid and incomplete questionnaires were excluded. The childhood trauma questionnaire has seven reverse-scored items, five of which constitute the subscale of emotional neglect (EN). Based on the comparison between the scores of reverse-scored items and the forward-scored items, we can better exclude invalid questionnaires (21). The questionnaires included general demographic characteristics (age, gender, BMI, education level, income level), childhood trauma, mental health (sleep quality, depressive symptoms, and COVID-19 related traumatic stress symptoms), and mental disorders (anxiety, depression, bipolar disorder, and schizophrenia) diagnosed by psychiatrists.

### Measures

The education was divided into nine levels, including illiteracy (1), primary school (2), middle school (3), vocational high school (4), senior high school (5), junior college (6), bachelor's degree (7), master's degree (8), and doctor's degree/Ph.D. (9). The income levels were collected as self-reported incomes compared with those of local people. It was divided into five categories: 1 = Low income level; 2 = Low-Middle income level; 3 = Middle income level; 4 = Middle-High income level; and 5 = High income level. Education and income level were covariates in the subsequent hierarchical linear regression analysis and mediation analysis.

### Childhood Trauma Questionnaires

Childhood trauma questionnaires-short form (CTQ-SF) was adopted to assess participants' experience from emotional abuse (EA), physical abuse (PA), sexual abuse (SA), EN, and physical neglect (PN) before 16 years old (21). A five-point Likert scale (1 = not at all, 5 = very often) was used to indicate the trauma severity about certain events or situations occurring during the childhood. Childhood trauma questionnaires-short form consisted of 25 clinical items and 3 validity items. The 25 clinical items incorporated five dimensions: EA, PA, SA, EN, and PN. Sum score of five subscales were CTQ total score ranging from 25 to 125, which indicated the severity of trauma exposure during childhood. Chinese version of CTQ-SF has good reliability and validity among Chinese undergraduates and depressive samples (22).

### The Pittsburgh Sleep Quality Index Questionnaire

Pittsburgh Sleep Quality Index questionnaire, a self-rated questionnaire, was used to assess sleep quality and disturbances over 1 month (23). This scale consisted of 19 individual items which generate seven component scores: subjective sleep quality, sleep latency, sleep duration, sleep efficiency, sleep disturbances, use of sedative-hypnotic drugs, and daytime dysfunction. The sum of the scores for these seven components ranges from 0 to 21, with a score ≤ 5, 6–10, 11–15, and 16–21 indicating very good, fairly good, fairly poor, and very poor sleep quality, respectively.

### Impact of Event Scale-Revised

Impact of Event Scale-Revised (IES-R), a self-administered questionnaire, was used to assess psychological impact of the COVID-19 epidemic on all participants. The IES-R included 22 items and consisted of three components: avoidance, intrusion, and hyperarousal. The total IES-R score was the sum score of the three components, with scores 0–23, 24–32, 33–36, and >37 indicating normal, mild, moderate, and severe psychological impact, respectively (24). The Chinese version of IES-R has been well-validated in the Chinese population, with high Cronbach's alpha coefficients for subscales [0.89 (intrusion), 0.85 (avoidance), and 0.83 (hyperarousal)] (25).

### Depression, Anxiety, and Stress Scale

The Depression, Anxiety, and Stress Scale (DASS-21) was used to evaluate individual's mental health status in the last week. Depression, Anxiety, and Stress Scale included 21 items and could be divided into subscales of depressive, anxiety, and stress symptoms. The DASS-21 subscales were scored as follows: normal (0–9), mild (10–13), moderate (14–20), severe (21–27), and extremely severe (28+) for depression; normal (0–7), mild (8,9), moderate (10–14), severe (15–19), and extremely severe (20+) for anxiety; normal (0–14), mild (15–18), moderate (19–25), severe (26–33), and extremely severe (34+) for stress (26). The Cronbach's alpha for Chinese version of DASS-21 was 0.95 for total scale, demonstrating a good internal consistency in the assessment of mental health in Chinese population (27).

### Statistical Analysis

Pearson correlation analysis was conducted to calculate correlation coefficients between the total scale and five subscales of CTQ, perceived psychological impact of COVID-19 pandemic, and adverse mental health symptoms. Then, we applied a hierarchical linear regression to estimate the role of CTQ toward DASS-21, IES-R, and PSQI global sleep quality by sequentially adding predictors into two blocks within each model. The variables were added into models *via* the following steps: Step 1: input the demographic characteristics of age, sex, BMI, and education level; Step 2: add CTQ. Finally, a series mediation analysis was carried out to examine the mediated effects of perceived impact of the COVID-19 pandemic and PSQI global sleep quality in the relationship between childhood trauma and mental health status. The series mediation analysis was conducted by Process 3.5 for SPSS version 24.0 (model 6). The significance levels of direct, indirect, and mediated effects among the four factors [i.e., childhood trauma (X), the psychological impact of events (M1), PSQI global sleep quality (M2), and mental health parameters (Y)] were determined as two-tailed *p*-values < 0.05, such figure being considered statistically significant in all other tests of this study. All continuous variables were standardized and then included in regression and mediation analyses performed on SPSS 24.0.

## Results

### Demographic Information, Childhood Trauma, COVID-19 Related Psychological Impact, Sleep Quality, and Mental Health Status

After excluding invalid and incomplete questionnaires, 817 (86.18%) of 948 participants were enrolled in this study. The mean age (± SD) was 27.77 (± 8.68) years old. The average education level and income were junior college and Low-medium income level, respectively. The mean scores of CTQ total scale and subscales of EA, PA, SA, EN, and PN were 37.03, 6.92, 6.12, 5.55, 9.77, and 8.66, respectively. The mean scores of DASS-21 total scale and subscales of anxiety, depression, and stress were 14.56, 4.15, 4.96, and 5.38, respectively. The mean scores of IES-R total scale and subscales of avoidance, intrusion, and hyperarousal were 9.04, 3.23, 3.40, and 2.40, respectively. The mean score of PSQI total score was 4.27. Of all subjects, there were 19.51, 20.73, and 9.76% individuals reporting mild to extremely severe anxiety, depressive, and stress symptoms, respectively. There were 25 (3.06%), 38 (4.45%), 10 (1.22%), and 6 (0.73%) subjects who were diagnosed by psychiatrists as depression, anxiety, bipolar disorder, and schizophrenia, respectively (Table 1).

**Table 1:** Descriptive statistics, *N* = 817.

	**Mean**	**SD**	**Min**	**Max**
Age	27.77	8.68	16	59
Gender (M/F)	431/386			
BMI	22.22	3.46	14.43	37.30
EDU level	6.27	1.52	1	9
Income level	2.44	0.85	1	5
**Childhood Trauma Questionnaire (CTQ)**				
Mean CTQ score	37.03	11.15	25	97
EA	6.92	2.89	5	25
PA	6.12	2.55	5	25
SA	5.55	1.82	5	25
EN	9.77	4.67	5	24
PN	8.66	3.42	5	21
**Depression, Anxiety, Stress and Stress Scale-21 (DASS-21)**				
Mean DASS-21 total score	14.56	20.78	0	126
DASS-21 (anxiety)	4.15	6.47	0	42
No (0–7)	657 (80.42)			
Mild (8,9)	35 (4.28)			
Moderate (10–14)	76 (9.30)			
Severe (15–19)	18 (2.20)			
Extremely severe (20+)	31 (3.79)			
Mean DASS-21 depression score	4.96	7.61	0	42
No (0–9)	647 (79.19)			
Mild (10–13)	66 (8.08)			
Moderate (14–20)	67 (8.20)			
Severe (21–27)	14 (1.71)			
Extremely severe (28+)	23 (2.82)			
Mean DASS-21 stress score	5.38	7.26	0	42
No (0–14)	737 (90.21)			
Mild (15–18)	27 (3.30)			
Moderate (19–25)	30 (3.67)			
Severe (26–33)	16 (1.96)			
Extremely severe (34+)	7 (0.86)			
**Impact of event scale-revised (IES-R)**				
Mean IES-R score	9.04	10.49	0	88
Mean IES-R avoidance score	3.23	4.19	0	32
Mean IES-R intrusion score	3.40	4.13	0	32
Mean IES-R hyperarousal score	2.40	3.39	0	24
PSQI global sleep quality score	4.27	2.95	0	17
**Self-reported diagnosed mental disorders by psychiatrists**				
Depression, *n* (%)	25 (3.06)			
Anxiety, *n* (%)	38 (4.65)			
Bipolar disorder, *n* (%)	10 (1.22)			
Schizophrenia, *n* (%)	6 (0.73)			

*BMI, body mass index; CTQ, childhood trauma questionnaires; DASS-21, depression, anxiety, stress and stress scale-21; EA, emotional abuse; EDU, education; EN, emotional neglect; IES-R, impact of event scale-revised; PA, physical abuse; PN, physical neglect; PSQI, Pittsburgh sleep quality index; SA, sexual abuse*.

### Correlations Among Childhood Trauma, COVID-19 Related Psychological Impact, Sleep Quality, and Mental Health Status

Correlations of total scale scores of CTQ, PSQI sleep quality, DASS-21, and IES-R and subscales of those are displayed in Table 2. After Bonferroni correction, all the variables were significantly correlated (*p* < 0.05) except for SA with DASS-21 total scale, stress and depression subscales, IES-R total scale, avoidance, intrusion, and hyperarousal subscales, and PSQI total scale (*p* > 0.05); except for EN with IES-R avoidance subscale (*p* > 0.05); and except for PN with IES-R avoidance subscale (*p* > 0.05). The total score of childhood trauma was positively and significantly associated with the perceived psychological impact of the pandemic, sleep quality, as well as DASS-21 anxiety, depression, and stress scores (*p* < 0.001). The PSQI total scale score was positively and significantly associated with the psychological impact of the pandemic, DASS-21 total score, and subscales of anxiety, depression, and stress scores (*p* < 0.001). Moreover, the IES-R total scale score was positively and significantly associated with DASS-21 total scores and all subscale scores (*p* < 0.001).

**Table 2:** Bivariate Pearson correlations with Bonferroni correction between main variables of interest.

	**1**	**2**	**3**	**4**	**5**	**6**	**7**	**8**	**9**	**10**	**11**	**12**	**13**	**14**	**15**
CTQ	1														
EA	0.772^c^	1													
PA	0.671^c^	0.659^c^	1												
SA	0.551^c^	0.483^c^	0.490^c^	1											
EN	0.778^c^	0.398^c^	0.236^c^	0.173^c^	1										
PN	0.753^c^	0.379^c^	0.300^c^	0.256^c^	0.568^c^	1									
DASS-21-T	0.353^c^	0.417^c^	0.228^c^	0.121	0.286^c^	0.174^c^	1								
DASS-21-A	0.347^c^	0.418^c^	0.245^c^	0.137^b^	0.264^c^	0.162^c^	0.959^c^	1							
DASS-21-D	0.359^c^	0.414^c^	0.213^c^	0.113	0.302^c^	0.187^c^	0.959^c^	0.879^c^	1						
DASS-21-S	0.313^c^	0.374^c^	0.202^c^	0.100	0.256^c^	0.152^b^	0.963^c^	0.894^c^	0.876^c^	1					
IES-R-T	0.240^c^	0.272^c^	0.166^c^	0.104	0.160^c^	0.157^c^	0.601^c^	0.608^c^	0.521^c^	0.607^c^	1				
IES-R-A	0.160^c^	0.210^c^	0.125^a^	0.077	0.078	0.105	0.477^c^	0.480^c^	0.417^c^	0.480^c^	0.890^c^	1			
IES-R-H	0.301^c^	0.327^c^	0.192^c^	0.111	0.235^c^	0.180^c^	0.673^c^	0.677^c^	0.593^c^	0.673^c^	0.864^c^	0.653^c^	1		
IES-R-I	0.201^c^	0.209^c^	0.138^c^	0.095	0.132^a^	0.144^b^	0.490^c^	0.501^c^	0.415^c^	0.501^c^	0.917^c^	0.734^c^	0.711^c^	1	
PSQI	0.215^c^	0.250^c^	0.095	0.073	0.191^c^	0.119	0.549^c^	0.521^c^	0.523^c^	0.537^c^	0.393^c^	0.269^c^	0.487^c^	0.325^c^	1

*CTQ, childhood trauma questionnaire; DASS-21, depression, anxiety, stress and stress scale-21; DASS-21-A, DASS-21 anxiety score; DASS-21-D, DASS-21 depression score; DASS-21-T-S, DASS-21 stress score; DASS-21-T, DASS-21 total score; EA, emotional abuse; EN, emotional neglect; IES-R, impact of event scale-revised; IES-R-A, IES-R avoidance score; IES-R-H, IES-R hyperarousal score; IES-R-I, IES-R intrusion score; IES-R-T, IES-R total score; PA, physical abuse; PN, physical neglect; PSQI, Pittsburgh sleep quality index; SA, sexual abuse*.

a *p < 0.05;*

b *p < 0.01;*

c *p < 0.001*.

### The Hierarchical Linear Regression Analysis of Psychological Impact Related to COVID-19, Mental Status, and Sleep Quality

Table 3 shows the results of hierarchical linear regression analysis between childhood trauma and self-reported health status in all respondents, with adjustment to age, gender, BMI, and education level. In the hierarchical linear regression analysis, socioeconomic status, such as income level and educational level of parent(s), was incorporated into the model, but such model was not significant, thus excluding the socioeconomic factors from covariates in step 2. The final regression model (model 2) explained 17.3, 8.3, and 9.5% of the total variance in DASS-21, IES-R, and PSQI total scale scores, respectively.

**Table 3:** Hierarchical regressions between CTQ total scale scores and DASS-21, IES-R, and PSQI global sleep quality scores.

	**DASS-21**			**IES-R**			**PSQI global sleep quality**		
	**β**	***SE***	***P***	**β**	***SE***	***P***	**β**	***SE***	***P***
**Step 1**	***R***^**2**^ **=** **0.044**, ***F*** **=** **8.030**, ***p*** **=** **0.000**			***R***^**2**^ **=** **0.021**, ***F*** **=** **4.039**, ***p*** **=** **0.003**			***R***^**2**^ **=** **0.040**, ***F*** **=** **8.743**, ***p*** **=** **0.000**		
Age	−0.085	0.035	0.014	−0.029	0.041	0.471	0.009	0.037	0.810
Sex	0.220	0.087	0.000	0.151	0.082	0.000	0.106	0.079	0.007
BMI	0.075	0.043	0.086	0.113	0.041	0.006	−0.014	0.039	0.727
Education	0.026	0.024	0.480	−0.009	0.024	0.812	0.150	0.023	0.000
**Step 2**	***R***^**2**^ **=** **0.173**, **Δ*****R***^**2**^ **=** **0.129**, ***F*** **=** **126.092**, ***p*** **=** **0.000**			***R***^**2**^ **=** **0.083**, **Δ*****R***^**2**^ **=** **0.062**, ***F*** **=** **54.771**, ***p*** **=** **0.000**			***R***^**2**^ **=** **0.095**, **Δ*****R***^**2**^ **=** **0.055**, ***F*** **=** **48.733**, ***p*** **=** **0.000**		
Age	−0.083	0.032	0.009	−0.028	0.039	0.475	0.010	0.035	0.776
Sex	0.189	0.080	0.000	0.129	0.078	0.001	0.086	0.076	0.024
BMI	0.076	0.042	0.073	0.114	0.040	0.004	−0.013	0.039	0.738
Education	0.064	0.022	0.058	0.018	0.023	0.603	0.174	0.022	0.000
CTQ	0.362	0.046	0.000	0.251	0.045	0.000	0.236	0.038	0.000

*CTQ, childhood trauma questionnaire; DASS-21, depression, anxiety, stress and stress scale-21; IES-R, impact of event scale-revised; PSQI, Pittsburgh sleep quality index; SE, standard error*.

Total score of childhood trauma was a significant predictor for COVID-19 related psychological impact, mental status, and PSQI global sleep quality (*p* < 0.001).

### Series Mediation Effects of COVID-19 Related Psychological Impact and Sleep Quality Between Childhood Trauma and Mental Health Status

**Figure 1:**
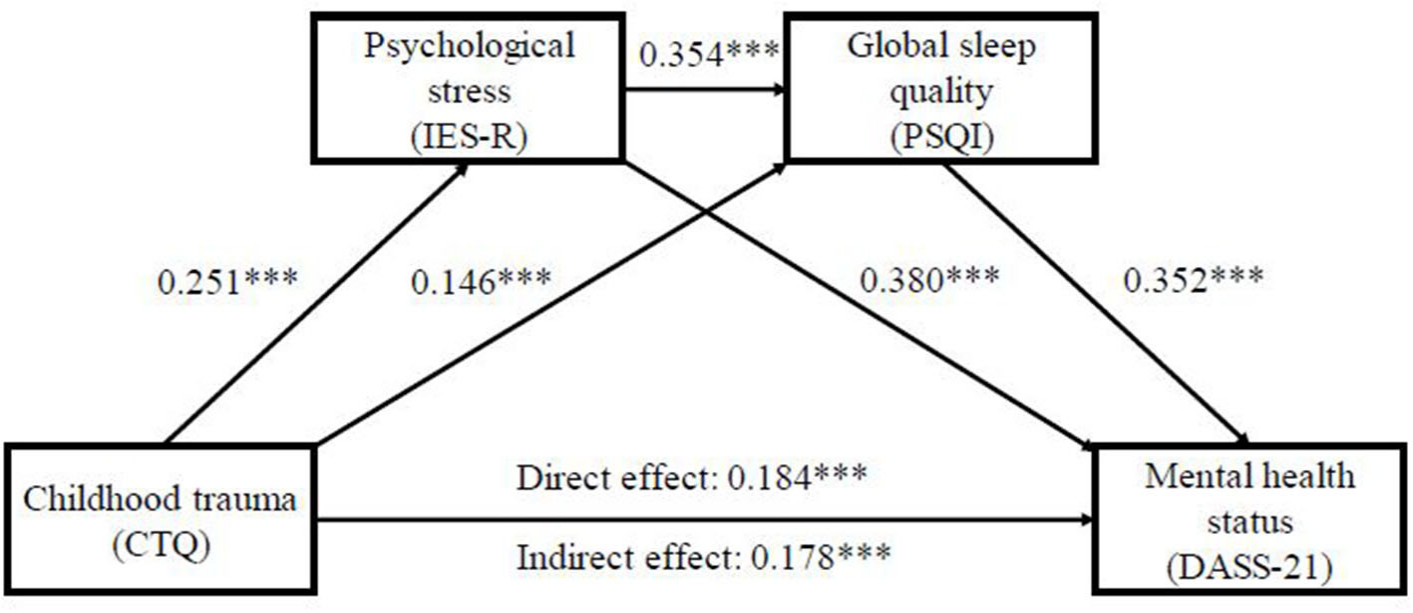
Series mediation effects of psychological stress and sleep quality between childhood trauma and mental health status. CTQ, childhood trauma questionnaire; DASS-21, depression, anxiety, stress and stress scale-21; IES-R, impact of event scale-revised; PSQI, Pittsburgh sleep quality index. ***p < 0.001.

Figure 1 shows the series mediating effect of psychological impact related to COVID-19 pandemic and PSQI global sleep quality in the association between childhood trauma and self-reported mental health status. All the paths in this model were significant (*p* < 0.001). First, the direct effect of childhood trauma on mental health status was explored, and the results showed that the model fitted well with *R*^2^ = 0.173, *F* = 33.840, and *p* < 0.001. Specifically, childhood trauma can directly and positively predict mental health status (β = 0.362, *p* < 0.001). Second, considering childhood trauma as a independent variable, mental health status as the dependent variable, and psychological impact related to COVID-19 pandemic and PSQI global sleep quality as mediating variables, model (childhood trauma → psychological impact → sleep quality → mental health, see Figure 1) was thereby established. The mediation model exhibited acceptable goodness of fit (*R*^2^ = 0.504, *F* = 116.845, *p* < 0.001). This study further conducted the bootstrapping method for 5,000 times to test the significance of the mediating effect. The mediating effect was significant if the 95% confidence interval did not include 0. The mediating effects of psychological impact and sleep quality on the association between childhood trauma and mental health status were significant (95% confidence intervals were 0.095 [0.059, 0.136] and 0.051 [0.028, 0.078], respectively), and the series mediating effect was also significant 0.031 [0.020, 0.045]. The total mediation effect and the direct effect between childhood trauma and mental health were 0.178 [0.128, 0.228] and 0.184 [0.133, 0.235], respectively (Tables 4, 5). Overall, the association between childhood trauma and mental health status was partly mediated by psychological impact and sleep quality (proportion explained 49.17%).

**Table 4:** Results of mediation analysis.

	**Fit index**							**%95 CI**	
	***R***	***R^**2**^***	***F***	***B***	***SE***	***T***	***p***	**LLCI**	**ULCI**
**Dependent variable: IES-R**									
CTQ	0.289	0.083	146.88	0.251	0.034	7.400	0.000	0.185	0.318
Age				−0.028	0.036	−0.775	0.439	−0.099	0.043
Sex				0.260	0.076	3.44	0.000	0.111	0.408
BMI				0.114	0.038	3.02	0.003	0.040	0.188
Education				0.012	0.023	0.521	0.602	−0.033	0.056
Constant				−0.456	0.171	2.665	0.008	−0.792	−0.120
**Dependent variable: PSQI global sleep quality**									
CTQ	0.458	0.210	35.697	0.162	0.033	4.494	0.000	0.082	0.210
IES-R				0.354	0.033	10.846	0.000	0.290	0.418
Age				0.020	0.034	0.593	0.553	−0.046	0.086
Sex				0.080	0.071	1.128	0.260	−0.059	0.218
BMI				−0.053	0.035	1.514	0.131	−0.122	0.016
Education				0.110	0.021	5.253	0.000	0.069	0.152
Constant				−0.811	0.159	5.090	0.000	−1.123	−0.498
**Dependent variable: DASS-21**									
CTQ	0.710	0.504	116.845	0.184	0.026	7.029	0.000	0.133	0.235
IES-R				0.380	0.028	13.692	0.000	0.325	0.434
PSQI				0.352	0.028	12.576	0.000	0.297	0.407
Age				−0.078	0.027	2.921	0.004	−0.131	−0.026
Sex				0.221	0.056	3.945	0.000	0.111	0.331
BMI				0.037	0.028	1.328	0.185	−0.018	0.092
Education				−0.003	0.017	−0.154	0.878	−0.036	0.031
Constant				−0.307	0.129	2.386	0.017	−0.560	−0.054

*B = unstandardized coefficient (the continuous variables included in the mediation analysis were standardized). BMI, body mass index; CTQ, childhood trauma questionnaire; DASS-21, depression, anxiety, stress and stress scale-21; IES-R, impact of event scale-revised; PSQI, Pittsburgh sleep quality index; SE, standard error*.

**Table 5:** Results of the series mediating effects after bootstrapping test.

	**Indirect Effect**			
	**β**	**Se**	**LLCI**	**ULCI**
CTQ → IES-R → DASS-21				
	0.095	0.020	0.059	0.136
CTQ → PSQI → DASS-21				
	0.051	0.013	0.028	0.078
CTQ → IES-R → PSQI → DASS-21				
	0.031	0.006	0.020	0.045
Total mediation effects				
	0.178	0.026	0.128	0.228

*CTQ, childhood trauma questionnaire; DASS-21, depression, anxiety, stress and stress scale-21; IES-R, impact of event scale-revised; PSQI, Pittsburgh sleep quality index; SE: standard error*.

## Discussion

To our knowledge, this is the first study using series mediation model to examine whether the association between childhood trauma and mental health status could be partly explained by COVID-19 pandemic related psychological impact and sleep quality. There were two findings worth highlighting. First, childhood trauma was a risk factor for COVID-19 related psychological distress (avoidance, intrusion, and hyperarousal), sleep quality, and mental health status (depressive, anxiety, and stress symptoms). Moreover, after the effect of psychological impact related to COVID-19 pandemic was incorporated into the model, the impact of childhood trauma on mental health status was partly mediated by psychological distress for COVID-19 and global sleep quality.

The links between childhood trauma and poor sleep, depression, and anxiety were well-documented. One study found that severer childhood trauma was associated with poorer sleep health, including sleep quality, sleep efficiency, sleep duration, and daytime sleepiness (28). On the other hand, some cross-sectional studies found that childhood trauma was not only associated with depression and anxiety symptoms in the clinical samples and general population but also related to the onset and recurrence of depressive and anxiety disorders (29–31). Previous studies reported that dysfunction of HPA axis, cognitive emotion dysregulation, epigenetic regulation of the stress response, and abnormal change of brain structural and functional plasticity may create a barren climate for the development of mental health (16, 29, 32, 33).

This study highlighted that childhood trauma was associated with COVID-19 related psychological distress. A few studies strongly supported that severer childhood trauma prior to the COVID-19 pandemic predicted greater risks of occurring psychological symptoms (17–19, 34, 35). One prospective study identified that childhood adverse experiences increased the risk of both psychological distress (ORs = 2.00–2.66) and probable acute stress reaction (ORs = 2.23–3.10) (5). Research evidence suggested that childhood trauma was associated with increased vulnerability to the stressful effect of the COVID-19 outbreak. Several potential mechanisms could interpret the association between the two. First, stress-vulnerability model assumes that childhood trauma, as chronic stress in the early life, could lower one's threshold of tolerance to acute stress, such as the outbreak of COVID-19 (16). Second, systemic inflammation may be an important mediator between childhood maltreatment and mental health in adulthood. Adults with exposure to childhood maltreatment exhibited a stronger inflammatory response under a standardized psychosocial stressor (36). Third, childhood trauma may change neuroendocrine responses to stress and increase vulnerability to acute events. One study found that limbic-medial temporal lobe regions, including amygdala and hippocampus, were sensitized in individual's exposure to life trauma. These regions could accommodate stress regulation/regulate stress and HPA axis function, and increase risk for negative stress-related mental health (37).

The results of the mediating effect test reveal that childhood trauma can affect adults' mental health through multiple mediating effects exerted by psychological impact of COVID-19 pandemic and sleep quality. Psychological impact of COVID-19 pandemic played a mediating role in the association between childhood trauma and mental health. To put it in another way, childhood trauma can predict mental health not only in a direct way but also in an indirect way through COVID-19 related psychological impact and sleep quality. It was suggested by a previous study that insomnia symptoms partly mediate the relationship between childhood trauma and the severity of depressive and anxiety symptoms in patients with psychosis (20). This is also similar to the conclusion drew by John-Henderson that psychological stress mediated the association between childhood trauma and sleep quality (18). Childhood trauma is mainly related to the adaptive dimension of stress. This shows that people who experience severer childhood trauma are more likely to suffer a high degree of psychological impact. This result indicates that early intervention in children's exposure to trauma may reduce the occurrence of event-related trauma symptoms (38). Therefore, childhood trauma can lead to greater COVID-19 related psychological impact and, thus, poorer mental health status. The results of this study confirmed the series mediating roles of COVID-19 related psychological impact and PSQI global sleep quality in the association between childhood trauma and mental health.

There are several limitations in this study that should be noted. First, this study was based on self-reported questionnaires, which could lead to self-awareness and reporting bias. Second, due to the cross-sectional design in this study, the causal relationships between childhood trauma and mental health may be weakened. Third, the COVID-19 related changes of people's life (e.g., family conflicts and unemployment) were not included in our study. Therefore, the impact of these confounding factors on sleep quality and mental health could not be controlled. Fourth, the associations between childhood trauma and mental health are also affected by other mental disorders, which are not controlled in our analysis. Longitudinal design needs to specify the direction of the relationships between childhood trauma and mental health or experimentally observe childhood trauma to explore the resultant changes in mental health in the following research.

## Conclusion

This study found that childhood trauma was positively associated with COVID-19 related psychological impact (avoidance, intrusion, and hyperarousal), anxiety, depression, and stress symptoms. In addition, the results of the series mediation analysis indicated an underlying mechanism of the association between childhood trauma and mental health: severer childhood trauma was able to predict poorer mental health outcomes *via* COVID-19 pandemic related psychological impact and sleep quality. Early interventions should be implemented to raise public awareness of adverse consequences of childhood trauma, whether emotional or physical, and poor mental health *via* family and social strategies. Extra attention should be paid to individuals who are experiencing or have experienced trauma or bully in the past. In addition, psychiatrists should also take patients' history of childhood trauma into consideration when making treatment decisions/in clinical decision-making.

## Data Availability Statement

The original contributions presented in the study are included in the article/supplementary material, further inquiries can be directed to the corresponding author/s.

## Ethics Statement

The studies involving human participants were reviewed and approved by Sichuan University. Written informed consent for participation was not provided by the participants' legal guardians/next of kin because: Written informed consent is difficult to obtain because we collected data via Wechat online survey. Because the questionnaire was anonymous, we assumed that participants consented to participate in our study by returning the questionnaire.

## Author Contributions

MX, YT, and LZ contributed to the writing of this article and the statistical analysis. QW and TL led the whole study, including carrying out this study, and putting forward the study. MD, YW, YH, YL, and LX contributed to the data collection and statistical analysis. All authors contributed to editing the manuscript and have approved the final manuscript.

## Funding

This study was funded by the National Natural Science Foundation of China (grant no. 81771446 and 82171499).

## Conflict of Interest

The authors declare that the research was conducted in the absence of any commercial or financial relationships that could be construed as a potential conflict of interest.

## Publisher's Note

All claims expressed in this article are solely those of the authors and do not necessarily represent those of their affiliated organizations, or those of the publisher, the editors and the reviewers. Any product that may be evaluated in this article, or claim that may be made by its manufacturer, is not guaranteed or endorsed by the publisher.
